# Hodgkin’s Disease of Intestine

**Published:** 1916-06

**Authors:** 


### ABSTRACTS.

---

Have you ever felt that this department of the BUFFALO MEDICAL JOURNAL was lacking in abstracts of your specialty, or that such as were published lacked the expert judgement which you yourself could have exercised in selection and preparation? If not, you are more charitable than the editor has been to himself. If so, will you assist in abstracting from a few journals, and thus make this department more representative and more valuable? Incidentally, there are few forms of study more helpful to the worker than this.

---

**Hodgkin's Disease of Intestine.** L. M. Warfield and H. T. Kristjanson of Milwaukee, Johns Hopkins Hosp. Bull. Jan., report a case in a Bulgarian man aged 27, a laborer. Previous history negative, gradually increasing pain in stomach, with vomiting and diarrhoea, for about nine months. Mucous rales and later dullness at the bases of the lungs suggested tuberculosis but no evidence of it was found. Necropsy showed that death was due to acute peritonitis with perforation of jejunum. Diffuse and nodular thickening of intestine, enlarged mesenteric glands, splenic tumor (280 grams), slight enlargement of liver (1600). The authors accept Bunting & Yates's demonstration of the specific bacillus Hodgkini and found some bodies resembling bacilli, Gram-positive, but do not consider them positively demonstrated in their case. The microscopic appearances of the glands was typic. References are given to several other intestinal and gastro-intestinal cases.

